# Controlling Apomixis: Shared Features and Distinct Characteristics of Gene Regulation

**DOI:** 10.3390/genes11030329

**Published:** 2020-03-20

**Authors:** Anja Schmidt

**Affiliations:** Department of Biodiversity and Plant Systematics, Centre for Organismal Studies, Heidelberg University, D-69120 Heidelberg, Germany; anja.schmidt@cos.uni-heidelberg.de; Tel.: +49-6221-54-4683

**Keywords:** apomixis, evolution, germline, gene regulation, sporogenesis, plant reproduction, ribosome, RNA helicase, sexual development, stress response

## Abstract

In higher plants, sexual and asexual reproduction through seeds (apomixis) have evolved as alternative strategies. As apomixis leads to the formation of clonal offspring, its great potential for agricultural applications has long been recognized. However, the genetic basis and the molecular control underlying apomixis and its evolutionary origin are to date not fully understood. Both in sexual and apomictic plants, reproduction is tightly controlled by versatile mechanisms regulating gene expression, translation, and protein abundance and activity. Increasing evidence suggests that interrelated pathways including epigenetic regulation, cell-cycle control, hormonal pathways, and signal transduction processes are relevant for apomixis. Additional molecular mechanisms are being identified that involve the activity of DNA- and RNA-binding proteins, such as RNA helicases which are increasingly recognized as important regulators of reproduction. Together with other factors including non-coding RNAs, their association with ribosomes is likely to be relevant for the formation and specification of the apomictic reproductive lineage. Subsequent seed formation appears to involve an interplay of transcriptional activation and repression of developmental programs by epigenetic regulatory mechanisms. In this review, insights into the genetic basis and molecular control of apomixis are presented, also taking into account potential relations to environmental stress, and considering aspects of evolution.

## 1. Plant Reproduction Is Characterized by Developmental Flexibility Including Sexual and Asexual Formation of Seeds (Apomixis)

Reproduction is an elementary process in the life cycles of all living species. In order to accomplish successful reproduction, propagation and adaptation, land plants adapted versatile strategies marked by commendable developmental flexibility. Apart from vegetative reproduction giving rise to offspring directly from tissues of the dominant sporophytic generation, sexual reproduction through seeds and apomixis are also common strategies. In contrast to sexual reproduction, apomixis leads to the formation of clonal offspring fully maintaining the genetic constitution of the mother plant. As this would allow the fixation of advantageous and complex genotypes, it has an outstanding potential for crop seed production. However, although apomixis is phylogenetically distributed in all major groups of angiosperms and occurs in more than 400 species [1,2,3,4,5,6,7], it is largely absent in major crop species. Therefore, engineering of apomixis for harnessing in agriculture is a longstanding aim [8,9,10]. To fully accomplish this, a detailed understanding of the genetic basis and the molecular mechanisms governing apomixis will be a prerequisite. So far, despite longstanding interest and research on apomixis, the underlying gene regulatory programs and their evolutionary origins are not well understood.

In this review, the developmental processes of sexual plant reproduction and apomixis are briefly outlined. The focus lies on a comprehensive description of current knowledge about the genetic basis and gene regulatory processes governing apomixis in different species and distinct types of apomixis. Thereby, it is intended to also propose and discuss new aspects and views to stimulate the scientific discussion on the topic. In addition, aspects of evolution and potential involvement of environmental conditions and stress regulations in apomixis control are presented. Detailed descriptions on gene regulatory programs underlying sexual reproduction can be found in other recent reviews [11,12,13].

**Figure 1:**
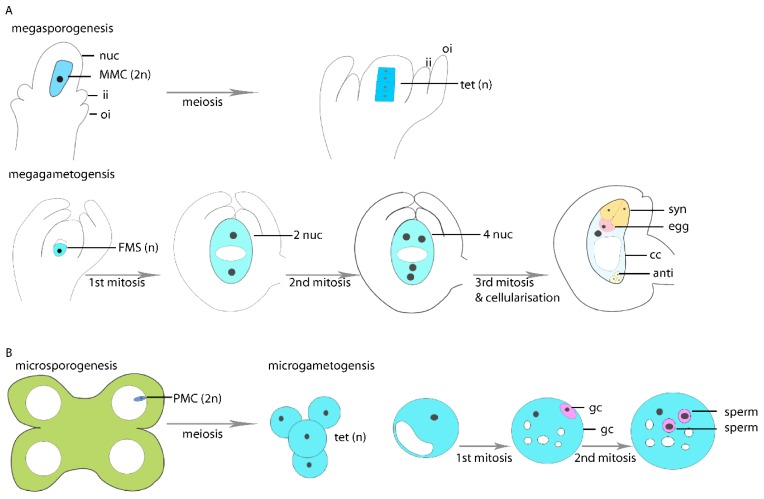
Development of the female (**A**) and male (**B**) reproductive lineages in sexual higher plants. (**A**) Formation of the female reproductive lineage initiates with the selection of a single diploid sporophytic cell in the nucellus (nuc) tissue of the ovule. This cell specifies as megaspore mother cell (MMC). Before meiosis of the MMC, the inner- and outer integuments of the ovule (ii and oi, respectively) are starting to grow. The MMC undergoes meiosis to give rise to a tetrad (tet) of haploid megaspores. Dependent on their position in the nucellus, three of the megaspores undergo apoptosis. Only the surviving functional megaspore (FMS) initiates gametogenesis. It undergoes three rounds of mitoses and cellularization to form the mature gametophyte harboring the two synergids (syn), the egg cell (egg), central cell (cc) and the antipodals (anti). (**B**) Formation of the male reproductive lineage initiates with selection of a single sporophytic cell, which is the pollen mother cell (PMC) that is committed to meiosis. Each of the four microspores of the tetrad (tet) survives and develops into a mature pollen by two mitotic divisions. During pollen mitosis I a generative cell (gc) engulfed in the vegetative cell (vc) is formed. During pollen mitosis II the generative cell divides to give rise to two haploid sperm cells.

From a developmental perspective, sexual reproduction and apomixis are related processes. In both cases, the female and male reproductive lineages (germlines) form in the reproductive tissues of the flower. These are the female ovules developing enclosed in the pistil and the male anthers, respectively. Germline specification and development proceeds in two consecutive steps, with mega- and microsporogenesis being the formation of mega- and microspores from selected female megaspore mother cells (MMCs) or male pollen mother cells (PMCs), respectively (Figure 1). Subsequent gametogenesis denotes the development of the female and male gametophytes (Figure 1). In higher plants they are reduced to a few cells only. During sexual reproduction, typically single sporophytic cells in the ovule and anther tissues are selected as MMCs or PMCs and determined for meiotic fate. The MMC is specified in a specialized domain of the developing ovule referred to as nucellus (Figure 1A). After meiosis, three of the four megaspores that have been formed undergo apoptosis and only one functional megaspore (FMS) survives as the founder cell of the gametophytic lineage. In the majority of angiosperms, a Polygonum-type mature female gametophyte (embryo sac) is formed by three rounds of mitosis in a syncytium and subsequent cellularization [14]. The mature gametophyte comprises seven cells and four distinct and specialized cell types (Figure 1A): the two female gametes, which are the egg cell and central cell that give rise to the embryo and the nourishing endosperm upon double fertilization, two synergid cells important for pollen tube guidance and reception, and three antipodal cells potentially playing a role in nourishing the gametophyte. Unlike in the female reproductive lineage during pollen development, all four meiotically formed microspores survive (Figure 1B). They undergo a first asymmetric mitotic division (pollen mitosis I) to form a two celled pollen with a generative cell engulfed in the vegetative cell. During a second mitotic division (pollen mitosis II), the two sperm cells derive from the generative cell. 

**Figure 2:**
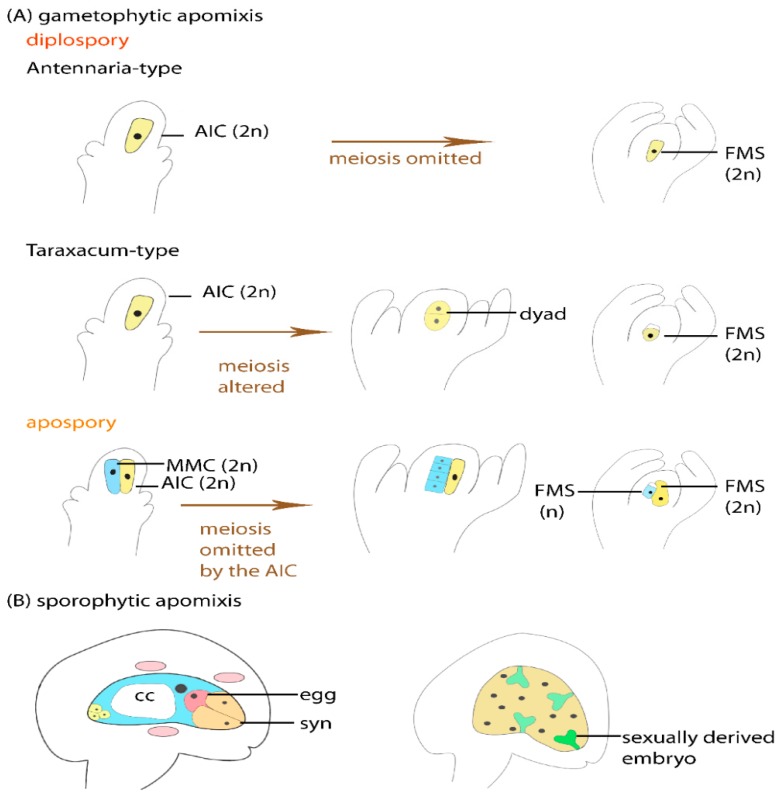
Major types of apomixis. (**A**) Different types of gametophytic apomixis are classified based on the origin and fate of the first cell of the germline lineage. In the Antennaria-type of diplospory the apomictic initial cell (AIC) directly specifies into an unreduced functional megaspore (FMS). In the Taraxacum-type of diplospory meiosis of the AIC is altered to give rise to a dyad of unreduced megaspores of which only one survives as the FMS. During apospory, an additional sporophytic cell in the ovule specifies adjacent to the sexual MMC. This cell omits meiosis to give rise to the FMS. While the sexual germline lineage typically gets repressed by the apomictic germline lineage, also the MMC can undergo meiosis resulting in the formation of two gametophytic lineages, one sexual, one apomictic, in the same ovule. (**B**) During sporophytic apomixis, the sexual gametophyte forms and additional sporophytic cells in the surrounding ovule tissues acquire the competency for embryogenesis (depicted in light red). After fertilization this typically leads to polyembryony, with the sexually derived embryo (dark green) and the somatic embryos (light green) competing for resources.

Compared to sexual reproduction, apomixis represents alterations of the developmental program. This concerns mainly a few steps during the formation and development of the female germline (Figure 2). It is commonly accepted that apomixis derived several times independently and that distinct types of apomixis are represented in higher plants [6,15]: First, sporophytic apomixis is distinguished from gametophytic apomixis, as the apomictic embryos either originate directly from sporophytic cells or from the egg cell formed in the gametophyte, respectively. Sporophytic apomixis, also known as adventitious embryony, is widespread throughout the plant kingdom and is particularly frequent e.g., in *Citrus* and *Orchidaceae* [6,16]. In sporophytic apomixis, one or more adventitious embryos derive from sporophytic cells of the nucellus, which is surrounding the sexually formed gametophyte (Figure 2B). Unlike sexual reproduction, which usually leads to the formation of a single embryo per seed, adventitious embryony is frequently marked by polyembryony [6]. Both the sexually derived embryo and its asexual siblings compete for resources of the endosperm (Figure 2B). While the formation of more than one embryo in a single seed is a feature of sporophytic apomixis, polyembryony can also occasionally result from gametophytic apomixis. However, polyembryony alone is not a clear indication for apomixis, as it also rarely occurs through sexual reproduction. This is the case in particular in gymnosperms, where apomictic reproduction appears to be largely absent [1]. 

Unlike through sporophytic apomixis, during gametophytic apomixis embryo and endosperm derive from the female gametes. To maintain the full genetic composition of the mother plant in the offspring, meiotic reduction and recombination need to be circumvented, as well as fertilization and thus the paternal contribution to the embryo. Developmental pathways of gametophytic apomixis are classified as diplospory or apospory [9,15,17] (Figure 2A). In diplosporous plants, the first cell of the female germline is an apomictic initial cell (AIC) developing in place of the MMC, but having a different fate. It undergoes a modified meiosis or it omits meiosis to give rise to an unreduced FMS (Figure 2A). Thereby, omission of meiosis and direct acquisition of gametophytic fate by the AIC holds true in Antennaria-type apomixis, while unreduced FMS are formed by restitution nucleus in Taraxacum-type and Ixeris-type of apomixis [10,18,19,20]. The Antennaria-type also referred to as mitotic diplospory has a wide systematic distribution [19]. In the Taraxacum-type of diplospory the MMC/AIC enters meiotic prophase I. As the chromosomes persist as univalent, this results in restitution nuclei remaining genetically identical to the sporophytic cells of the mother plant [21]. Also, the Ixeris-type of diplospory leads to formation of an unreduced FMS as consequence of restitution nucleus. In addition, 2n megaspores are formed in Allium-type of apomixis by premeiotic chromosome doubling [22]. Unlike in diplosporous apomicts, in aposporous apomicts, sexual and apomictic germlines initiate development in the same ovule (Figure 2A). One or several additional sporophytic cells adjacent to the sexual MMC directly give rise to the gametophytic lineage without intervening meiotic or mitotic divisions. Thereby, a competition of sexually and apomictically formed gametophytes can arise, or the development of the sexual gametophyte gets repressed by the apomictic germline lineage [22]. Independent of the developmental origin of the apomictic FMS, an embryo sac harboring egg cell and central cell is subsequently formed in all cases. To initiate seed development, the unfertilized egg cell then develops into an embryo parthenogenetically. The endosperm can form either autonomously without paternal contribution or by pseudogamy dependent on fertilization. Pseudogamy is prevalent in most apomicts. In contrast to the female germline, the male germline in apomicts may form either reduced or unreduced pollen [17].

Taken together, distinct developmental adaptations lead to formation of clonal offspring in the different apomictic systems. To date it is largely unclear if similar regulatory mechanisms are shared in all apomicts, if related mechanisms lead to apomixis, or if different pathways mediate apomictic reproduction in different taxa. Gaining insights into these questions is not only of interest from a scientific point of view, but also relevant for potential applications of apomixis. 

## 2. The Developmental Flexibility of Plant Reproduction might Hold Evolutionary Advantages

As outlined above, apomixis and sexual reproduction represent alternative strategies of reproduction that are developmentally related. From an evolutionary point of view, concepts on the origin and advantages of the different reproductive modes and their co-existence are still under debate and partially contradictory. Sexual reproduction is most commonly regarded as original mode of reproduction and asexuality as derived. However, this view has recently been questioned by new hypotheses proposing that both might represent evolutionary ancient concepts [18]. 

So far this puzzle has not unequivocally been resolved by phylogenetic analyses. While asexual reproduction is common in ~10% of species in fern, apomixis only occurs in less than 1% of angiosperms [1]. The representation of apomixis is broadly scattered in the angiosperms and has rapidly spread in the large families of *Poaceae*, *Asteraceae*, and *Rosaceae* [3]. Apart from the occurrence of apomixis in the genera *Draba*, *Erysimum*, and *Parrya*, in *Brassicaceae* apomixis is only represented in *Boechera* and the related genus *Phounicaulis* [23]. The absence of apomixis in *Amborella* as basal sister clade of angiosperms might rather indicate that it is derived, however, this alone cannot be taken as sufficient indication [6]. Moreover, the distinctiveness of the different types of apomixis like sporophytic and gametophytic apomixis, but also of apospory and diplospory, suggests that they represent non-homologous mechanisms that arose independently [24]. Consistently, apomictic lineages are commonly regarded as evolutionarily young. This is in line with the perception of apomixis being an evolutionary dead end due to an accumulation of deleterious mutations resulting from the absence of meiotic recombination [25]. Already in 1939 Darlington proposed that apomixis purely represents an escape from sterility that is often caused by polyploidization or hybridization typically associated with apomixis [26]. While polyploidy has long been regarded as a precondition for apomixis, the identification of diploid apomicts, e.g., in the genus *Boechera* has changed this view [27]. In *Boechera*, apomixis arose recurrently by hybridization and intra-specific crosses, and also outside *Boechera* evidence for hybrid origin is given for an increasing number of apomictic taxa [6,28].

Importantly, hybridization and polyploidization are likely to cause genome-wide effects resulting in alterations of gene regulation. It has long been hypothesized that apomixis derived by temporal and spatial deregulation of the gene regulatory pathway governing sexual reproduction [4,17]. Alternatively, or as a consequence of mutation accumulation in asexual species, apomixis might be caused by mutations in genes regulating sexual reproduction. However, this view is challenged by new findings and hypotheses [6,18]. In some systems like *Hieracium* and likely also in *Paspalum*, apomixis is superimposed on sexual reproduction and dominantly silencing sexual reproduction [22,29]. Interestingly, thereby the sexual pathway can be re-established, suggesting that the gene regulatory program underlying sexual reproduction remains intact. Thus, apomixis and sexual reproduction might indeed represent distinct and evolutionary ancient concepts [18]. In contrast to the classical view of apomixis as evolutionary dead end [25], recent studies suggest that apomixis might rather represent an evolutionary opportunity. Furthermore, sexual reproduction and apomixis might be regarded as competing strategies, as, for e.g., discussed for the facultative tetraploid aposporous apomict *Paspalum malacophyllum* [18,30]. Apomixis can be advantageous as it leads to the fixation of beneficial genotypes and it can be beneficial to overcome sterility and incompatibility effects [24]. Furthermore, in many systems studied to date, apomixis is largely facultative, setting the basis for a broad developmental flexibility. Together with the potential occurrence of reversions from apomictic reproduction to sexuality this allows to purge deleterious mutations from the genome [1,25,31,32,33]. 

The understanding of the evolutionary origin of apomixis will largely affect the routes of research taken to identify the gene regulatory basis of apomixis. An important aspect to elucidate is, if similar molecular mechanisms are underlying shared elementary features like parthenogenesis or the acquisition of gametophytic fate without preceding meiosis in different apomictic systems. From an evolutionary point of view, it remains astonishing that repeatedly all major components of apomixis could establish simultaneously in the different apomicts. Activation of any element of apomixis alone would have deleterious effects for the plants, e.g., apomeiosis without parthenogenesis would lead to polyploidization, parthenogenesis without preceding apomeiosis would lead to haploidization, and even the uncoupling of autonomous embryo development and endosperm development would prevent successful reproduction. Nevertheless, taking the apparent differences in the reproductive systems into account, it appears likely that also control mechanisms are diverse or similar mechanisms established by convergent evolution. Although the possibility cannot fully be ruled out that apomixis represents an ancient mechanism as an alternative to sexual reproduction, the non-homologues developmental pathways resulting in apomictic reproduction rather suggest independent origins. Different associated gene regulatory programs are likely to be required for certain developmental processes, in particular with respect to megasporogensis, which differs considerably in the different types of apomixis. While formation of an unreduced embryo sac by diplospory represents an alteration of the fate of the MMC/AIC, during apospory both the sexual and the apomictic germline lineage initiate their development so that a tight coordination and cross-talk between the two germline lineages is required. In aposporous *Hieracium* subgenus *Pilosella*, for example, the specification of the sexual MMC is preceding the formation of the aposporous initial cell, while the apomictic reproductive lineage subsequently suppresses further development of the sexual lineage [22]. Nevertheless, certain developmental flexibility and the formation of two gametophytes in the same ovule occurs in *Hieracium praeltum* and also in apomictic *Boechera*, providing the opportunity to reinforce the facultative nature of apomixis [33,34]. Also the occurrence of both, diplospory and apospory, demonstrates a striking flexibility of developmental concepts in *Boechera* [33]. Increasing attention is recently also given to the question, whether and how environmental factors are modulating the regulation of the reproductive programs or if they might even be sufficient to determine the reproductive mode.

## 3. Genetic Loci Linked to Apomixis Typically Represent Hemizygous Heterochromatic Regions 

Genetically in all taxa studied so far apomixis is heritable. This has been revealed by genetic analysis using an apomict as the male and a sexual plant as female parent [35]. Direct identification of the genes and genomic elements comprised on these apomixis linked loci however has proven difficult. This is because they are commonly recombination-suppressed and flanked by repetitive regions, interfering with sequencing approaches and map-based cloning [10,35,36,37,38,39,40,41]. In several species studied, apomixis linked loci represent chromosomal regions that largely diverged from corresponding sexual loci. They presumably originate from chromosomal rearrangements and transposable element activity consequently resulting in the frequently observed reduction or loss of recombination [42,43]. One locus is typically underlying each of the major components of apomixis, namely apomeiosis, parthenogenesis, and the developmental adaptations needed for endosperm formation [10,35,39,44]. 

Inheritance of diplospory as single dominant locus has been described for *Erigeron anuus* and for *Taraxacum officinale* [21,45]. In *Taraxacum*, two unlinked dominant loci control diplospory (DIPLOSPOROUS, DIP) and fertilization independent development of an embryo from the egg cell (PARTHENOGENESIS, PAR) [46]. The DIP locus thereby maps to the distal arm of one nucleolar organizer region (NOR) chromosome [46]. Unlike for most apomixis loci identified, recombination occurs in the hemizigous *Taraxacum* DIP locus that has been fine mapped to about 0.6 cM estimated to cover about 200–300 Kb [46,47]. Also, the apospory-specific genomic regions (ASGR) of *Pennisetum spamulatum* and *Cenchus ciliaris* are located on hemizygous heterochromatic regions on single chromosomes [48,49,50]. The apomixis-controlling locus (ACL) in *Paspalum simplex* is a single non-recombining hemizygous region [51]. In *Hieracium*, three loci have been identified to control apospory (LOSS OF APOMEIOSIS, LOA), parthenogenesis (LOSS OF PARTHENOGENSIS, LOP), and autonomous endosperm development (AutE) [52]. Also LOA was mapped to a recombination suppressed distal arm of a single chromosome and is surrounded by complex repeats and transposable elements that however are not essential for the function of the locus [53,54]. 

Further evidence for hemizygous and heterochromatic segments of chromosomes is given from investigations on *Boechera*, where the presence of the largely heterochromatic B-like chromosomes (Het and Del) has been observed in apomictic accessions by karyotype analyses [55,56,57,58]. Also in the closely related *Boecheraea* genus *Phoenicaulis* a largely heterochromatic Het chromosome is present in triploid and tetraploid cytotypes, unlike in diploid [23]. While it has been hypothesized that these chromosomes might be relevant for apomixis expression and in particular possible implications for diplospory have been discussed, transmission of a Het chromosome alone is not sufficient for apomixis to arise [23,59]. Interestingly, the Het chromosomes and heterochromatic chromosomal regions linked to apomixis resemble features of Y-chromosomes in animals and dioecious plants for sex determination with respect to typical accumulation of transposable elements and gene loss [29,46,60]. Therefore, it is tempting to speculate that similarly to well known mechanisms in Y-chromosomes, epigenetic regulatory mechanisms might be major driving forces in regulation of apomixis. Epigenetic regulatory mechanisms in general are involved in controlling gene activity by DNA methylation, introducing repressing or activating histone modifications, as well as modulation of overall chromosome structure.

### Genes Located on Apomixis Loci Suggest That Different Regulatory Pathways Are Involved in Controlling Apomixis

Despite the similar features presented by the apomixis linked loci so far investigated, knowledge about the genes encoded and their roles in controlling apomictic development is scarce to date. In order to confer successful reproduction, it is essential that all elements of apomixis and associated developmental processes take place in a coordinated manner. To allow this, these regions might include master or key regulatory genes activating a downstream cascade controlling all major aspects of apomixis, or many linked genes encoded on the apomixis linked loci might be required [27]. So far, different genes have been identified to be linked to the apomixis loci in different species (Table 1). Based on these findings, several distinct regulatory mechanisms appear to be involved in controlling apomixis. These include the activity of transcription factors, but also degradation of nucleobases and control of protein turnover, and the modulation of gene activity by mechanisms involving non-coding RNAs. In particular long non-coding RNAs including antisense RNAs are increasingly recognized as important players involved in the regulation of reproduction and a range of developmental decisions [61]. 

For apomeiosis, a small number of candidate genes from a few loci have been proposed: In *Hypericum perforatum* the Hypericum Apospory- (HAPPY-)locus is co-segregating with apospory but not with parthenogenesis [44] (Table 1). This locus contains a truncated allele of the homologue of *Arabidopsis thaliana ARIADNE7* (*ARI7*), encoding for an E3 ligase Ring-finger protein involved in regulatory processes and protein degradation [44]. Consistent with the dominant nature of the HAPPY-locus, its simplex constitution has been confirmed in tetraploid plants [44]. Recently, sequencing approaches allowed the annotation of 33 predicted genes located on the HAPPY-locus, 24 of which were expressed in pre-meiotic nucellus tissues of the ovule [62]. In *Boechera*, two different candidates have been identified for regulation of female and male apomeiosis. As a candidate for female apomeisis, the APOmixis Linked LOcus (*APOLLO*) gene has been identified that is higher expressed in apomictic ovules at apomeiosis as compared to sexual ovules [63,64] (Table 1). Likewise, comparative transcriptome analyses of anthers containing pollen mother cells in sexual and apomictic *Boechera* identified that the activity of *UPGRADE (UPG)* is correlated with apomixis [65]. While it is inviting to hypothesize that *APOLLO* and *UPG* are likely to be localized on the heterochromatic HET and DEL chromosomes, a direct proof has so far not been presented [66]. *APOLLO* encodes for an Aspartate Glutamate Aspartate Aspartate Histidine exonuclease and is heterozygous for apomixis specific alleles in apomicts [63]. These alleles contain 20-nucleotide polymorphisms in the 5’ untranslated region (5’ UTR) [63]. Interestingly, *UPG2* represents a long non-coding RNA that has been proposed as candidate for the formation of unreduced pollen [64,65] (Table 1). Further evidence for roles of long non-coding RNAs in the regulation of (apo)meiosis comes from *Paspalum notatum* [67]. In *P. notatum* a long non-coding RNA related to a gene encoding mitogen-activated protein kinase kinase kinase (N46) is linked to the ACR [67] (Table 1). N46 is named *QUI-GON JINN* (*QGJ*) as a member of the YODA family. It is not only differentially expressed in flowers from sexual as compared to apomictic plants, but also its downregulation mediates a reduction of the rate of aposporous embryo sac formation [67]. Taken together the investigations on different apomicts provide increasing evidence for different regulatory mechanisms to control aspects of apomeiosis. Importantly however, as molecular mechanisms involved in the control of apomeiosis, mainly changes in gene regulation appear to be important that are enforced e.g., by changes in regulatory elements and the activity of non-coding RNAs. These findings do not provide evidence for the idea that elements of apomixis might derive from genetic mutations in coding regions that lead to alterations in protein function. Nevertheless, future investigations will be needed to more comprehensively understand the regulatory processes controlling apomeiosis.

Interestingly, pathways including the activity of non-coding RNAs might also be involved in the regulation of parthenogenesis and endosperm formation in certain apomicts. From the ACL of *Paspalum simplex*, expression of antisense transcripts for three genes has been identified [51,68]. This has led to the hypothesis that the ACL region modulates epigenetic processes regulating parthenogenesis and endosperm formation. This is consistent with the finding that parthenogenesis is superimposed on sexual reproduction in this system and that DNA demethylation affects parthenogenesis but not apomeiosis [29,51]. In particular the homologue of subunit 3 of the ORIGIN RECOGNITION COMPLEX (ORC3), which is functional in sexual plants, is regulated by an apomixis specific antisense pseudogene [68] (Table 1). The precise regulation of *ORC3* activity in apomicts appears to be relevant for formation of functional endosperm with a ratio of maternal to paternal contributions alternating from 2n: 1n [68]. 

Unlike the regulatory mechanisms involving non-coding RNAs, from the ASGR of *Pennisetum* and *Cenchrus BABY BOOM* (*BBM*)-like genes have been identified as promising candidates for parthenogensis based on the similarities to *BBM* of *Brassica napus* [49,69] (Table 1). *BBM* and *BBM*-like genes belong to a family of transcription factors characterized by two conserved APETALA2 (AP2) binding domains and a bbm-1 domain with functional implications for somatic embryogenesis [70]. From studies in *A. thaliana* BBM acts upstream of major regulators of totipotency and embryonic identity [71]. Originally identified as a gene involved in controlling somatic embryogenesis in microspore cultures, embryo development from somatic cells of *A. thaliana* leaves can be triggered by expression of *Brassica napus BBM* [72]. This supports its strong potential for inducing the gene regulatory program relevant to acquire the competence for embryogenesis. Evidence for the functional importance of *ASGR-BBML* for parthenogenesis was further substantiated by the identification of a *C. ciliaris* recombinant that retained apospory but lost parthenogenesis along with the *BBML* containing fragment of the ASGR [69]. Also, when expressed under its native promoter and terminator, expression of *ASGR-BBML* in egg cells of *Pennisetum squamulatum* is sufficient to trigger parthenogenesis in sexual plants [73]. Furthermore, studies from apomictic *Brachiara decumbens* suggest the importance of *ASGR-BBML* genes for parthenogenesis in *Poaceae* [74]. A recent study supports the broader validity of *BBM* genes to trigger embryogenesis and parthenogenesis by demonstrating that even in *Oryza sativa* expression of *BBM1* in egg cells is sufficient to allow autonomous embryo development in the absence of fertilization [75]. Strikingly, *BBM1* as the gene triggering embryogenesis behaves as an imprinted gene in young embryos, as only the paternal but not the maternal allele is expressed [75]. Imprinting in general describes a control mechanism that allows activity of one parental allele, while the allele from the other parent is silenced due to epigenetic regulation. The mechanism outlined for controlling *BBM1* activity elegantly explains the requirement for fertilization for seed development during sexual reproduction. As so far implications of BBM and BBML for parthenogenesis have only been described in monocotyledons, to date evidence is lacking for a broader importance of this mechanisms to repress embryogenesis of the egg cell in the absence of fertilization also in dicotyledons. 

From studies of somatic embryogenesis in *Citrus*, another transcription factor has been proposed to be relevant for the regulatory control. The genomic locus linked to somatic embryogenesis has first been described to comprise ~380 kb and it could further be fine-mapped to a genomic region of 80 kb harboring the sequences of 11 genes [5,16]. From this region, *CiRKD1* is recognized as candidate gene for polyembryony and somatic embryogenesis [5,76] (Table 1). *RKD* genes encode RWP-RK domain-containing transcription factors. In *A. thaliana* the five members of the family are predominantly expressed in the egg apparatus (egg cell and synergid cells) and are important regulators of gametogenesis and acquisition of egg cell fate [77,78,79]. Interestingly, in the egg apparatus of the triploid apomict *Boechera gunnisoniana*, *RKD* genes are present only at low levels [80]. This suggests that the gene family might play a role in maintaining egg cell identity in the absence of fertilization during sexual reproduction. Studies on *Marchantia polymorpha* with only a single *RKD* homologue represented in the genome indeed support the evidence of RKD to be an evolutionary conserved factor in plants important to acquire egg cell identity and to keep the egg cell in a developmentally repressed state in the absence of fertilization [81,82]. The regulation of acquiring the competence for embryogenesis and to activate this program might be more complex in *Citrus*. In *Citrus* studies from satsuma mandarin have recently revealed the presence of two *CiRKD1* alleles with one of them containing a miniature inverted-repeat transposable element (MITE)-like insertion in the upstream region [76]. Increased expression of this allele in the tissues where somatic embryogenesis occurs was observed and antisense silencing of *CiRKD1* in transgenic sweet orange leads to loss of somatic embryogenesis [76]. Interestingly, like in the case of *BBM* and *BBML*, differences in activity and regulation of one (type of) transcription factor(s) appear to be sufficient to acquire the competence for embryogenesis and to allow embryogenesis from a sporophytic cell or the egg cell in the absence of fertilization. 

## 4. Transcriptional Analysis Identifies Genes Differentially Regulated during Sexual and Apomictic Reproduction

Genetic studies identified only few apomixis linked loci suggesting that a limited number of genes might be required for apomixis. However, transcriptional studies often suggest a more global deregulation of the gene regulatory program underlying sexual reproduction in apomicts. This discrepancy might potentially be explained by master regulators which control complex programs of gene activity. Transcriptional analyses to identify genes differentially expressed in sexual as compared to apomictic plants have been presented for a variety of species including *Pennisetum ciliare* and *Pennisetum glaucum* [83,84], *Panicum maximum* [85,86], *Poa pratensis* [87,88], *Brachiaria brizantha* [89,90], *Paspalum notatum* and *Paspalum simplex* [91,92,93,94], *Eragostris curvula* [95], *Medicago falcata* [96], *Boehmeria tricuspis* [97], *Hypericum perforatum* [62,98], *Hieracium* [99,100], *Boechera* [101,102,103], and also for *Citrus* [76] (Table 2). These studies provide evidence for temporal deregulation of the gene regulatory processes governing sexual reproduction in apomicts and identify large numbers of up to hundreds of genes to be differentially expressed. 

Given the large numbers of genes identified as differentially regulated, it remains difficult to identify the genes that are relevant for the determination of the reproductive mode or developmental processes governing apomictic reproduction. As most of the studies are based on ovule or floral tissues it is likely that a large fraction of the genes identified is differentially expressed in sporophytic tissues rather than in the developing reproductive lineages. The overabundance of sporophytic tissues in the samples can mask the regulatory profiles controlling germline formation and development. To overcome this difficulty, cell and tissue type-specific transcriptome analysis, i.e., by combining laser assisted microdissection (LAM) with microarray analysis or RNA-Seq have proven to be powerful approaches [104,105,106]. Novel insights have already been gained into the gene regulatory pathways governing the development of the sexual MMC and the cells of the mature female gametophyte in *A. thaliana* [79,105,106,107], and the corresponding cells in the related triploid apomict *Boechera gunnisoniana* [80] (Table 2). Tissue type-specific transcriptome analysis targeting AIC/MMC and surrounding nucellus tissues furthermore allowed comparative analyses of gene expression and pathways relevant for megasporogenesis in different sexual as compared to apomictic *Boechera* accessions and in sexual versus aposporous *Hypericum perforatum* [62,103] (Table 2). LAM in combination with RNA-Seq recently has also shed light onto the cell type specification of the aposporous initial cell (AIC) as compared to early developing embryo sacs and somatic ovule tissues in *Hieracium praealtum*. These studies suggest advanced acquisition of gametophytic fate by the AIC [108,109] (Table 2). 

**Figure 3:**
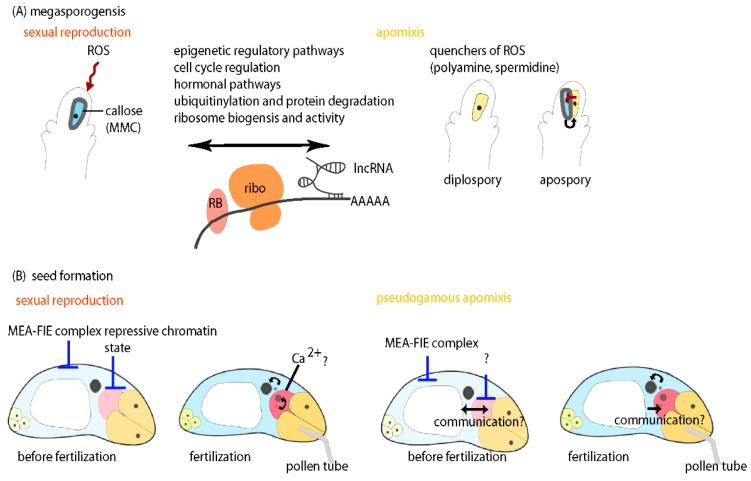
Molecular mechanisms differentially regulated during sexual reproduction and apomixis. (**A**) During megasporogenesis players in several pathways are differentially regulated implementing control of gene and protein activity. This likely involves also the activity of specialized ribosomes (ribo) in conjunction with RNA binding proteins (RB) like RNA helicases and products of non-canonical open reading frames including long non-coding (lnc) RNAs. Also stress and stress response appear to differentially affect megasporogensis in the different reproductive modes. During meiosis in sexual reproduction the MMC is enclosed by callose potentially as a response to reactive oxygen species (ROS). In contrast in apomicts high activity of polyamine biosynthesis and spermidine metabolism allows quenching of ROS. Furthermore, diplospory involves alterations in the meiotic program, while during apospory communication between the sexual and apomictic germline is required. (**B**) During sexual reproduction proliferation of the female gametes is repressed in the absence of fertilization by the activity of the MEA-FIE PRC2 complex in the central cell and a repressive chromatin state in the egg cell. Double fertilization initiates seed formation involving fusion of the two sperm cells with each of the female gametes. Likely rise in Ca^2+^-levels is involved in activation of the egg cell. In addition, in pseudogamous apomicts both female gametes need to remain repressed in the absence of fertilization of the central cell. Only the central cell nucleus fuses with sperm nucleus. Communication between the egg- and central cell is required to coordinate development.

Consistently, comparative transcriptome analysis in sexual and apomictic *Boechera* [103], *Bohemeria tricuspis* [97], and *Hypericum perforatum* [62] suggest that differential activity of genes involved in cell-cycle regulation, hormonal pathways, signal transduction, ubiquitinylation and protein degradation, and epigenetic regulatory pathway are involved in determining and sustaining megasporogenesis in either reproductive mode (Figure 3). To narrow down the number of candidate genes and to disentangle part of the effects of ploidy and species differences, differential expression analysis was recently applied to compare four apomictic versus two sexual *Boechera* accessions [103]. Thereby LAM and RNA-Seq have been combined to analyze gene expression in reproductive nucellus tissues harboring the AIC or MMC [103]. This has identified 45 genes to be consistently differentially expressed in all samples from sexual as compared to apomictic accessions [103]. This study supports the importance of genes involved in cell-cycle regulation, protein degradation and hormonal pathways for distinguishing sexual from apomictic reproduction, and also suggests functions related to stress and redox regulation to be relevant [103] (Figure 3). Taken together, evidence for the involvement of these pathways in regulation of megasporogenesis is consistently given from different apomicts. Apart from these investigations, additional transcriptional studies focused on mature gametophytes in sexual and apomictic plants and the transition to early stages of seed development would be beneficial to allow a more comprehensive understanding of the gene regulatory processes distinguishing apomixis from sexual reproduction. 

## 5. Different Layers of Regulation Are in Place to Control Development during Sexual and Apomictic Megasporogenesis

From the different apomicts a number of candidate genes for apomeiosis which are linked to apomixis loci have been determined (Table 1). As these genes are involved in diverse regulatory pathways they might be relevant for several aspects of development during megasporogenesis in the different types of apomixis. Thereby, potentially similar genes and molecular mechanisms might be required for the determination of the reproductive mode. However, complex regulatory programs should be in place controlling the distinct developmental programs associated with sexual and apomictic reproduction. To regulate megasporogenesis in diplosporous and aposporous apomicts, this should involve the determination of meiotic versus mitotic fate, the acquisition of germline identity, and the control and cell-cell communication to decide how many cells per ovule can make this fate transitions. In contrast for parthenogenesis, certain transcription factors appear to be crucial to acquire embryonic potency and to activate the regulatory program underlying embryogenesis.

### 5.1. Specialized Ribosomes and Associated Factors Emerge as Novel Players in Gene Regulation

Both in sexual plants and apomicts, germline formation and development requires complex and tight regulatory systems [12,105,116]. Different molecular machineries are in place to control gene expression and translation, protein activity and turnover, and cell-cell communication. Especially during megasporogenesis and preparation of the MMC/AIC for (apo)meiosis, processes related to translation and ribosome biogenesis are enriched both in sexual *A. thaliana* and apomictic *Boechera* [103,107]. Further evidence for the importance of ribosome biogenesis and function, and nucleosome assembly for megasporogenesis in apomicts comes from transcriptome analysis of *Hieracium* subgenus *Pilosellum*, as related functions are enriched in the AIC as compared to developing embryo sacs [109]. While it has long been noticed that a cycle of ribosome degradation and reassembly is associated with plant meiosis [117], the relevance of this processes for sexual and apomictic reproduction has not been described in detail to date. Nevertheless, functions related to ribosome assembly are over-represented in transcripts from the ASGR of *Pennisetum squamulatum*, suggesting that the control of ribosome activity is playing a role for apomixis [118]. A further hint in this direction is presented from the localization of DIP locus on a NOR chromosome in *Taraxacum* [46].

While ribosomes are more historically thought to have constitutive functions in mRNA translation, recently the notion has emerged of specialized ribosomes as an additional layer of gene regulation that has so far been largely overlooked. It might represent a mechanism to regulate a switch of developmental fate such as the determination of meiosis or apomeiosis by co-regulation of a larger number of relevant target genes. The ribosome based regulatory machinery involves specific mRNA regulatory elements such as internal ribosome entry sites which are mostly located in the 5’ UTR regions of mRNAs [119]. Recent findings also point towards the importance of non-canonical open reading frames (ORFs) including non-coding RNAs in the regulation of ribosome biogenesis and function [120,121] (Figure 3). While estimates suggest that e.g., in *A. thaliana* up to 80–90% of the genome is transcribed at least at a certain developmental time point, less than half of these transcripts appear to be coding proteins or peptides [122]. The non-protein coding RNAs comprise housekeeping RNAs like ribosomal RNAs, tRNAs, small nuclear and nucleolar RNAs, and small RNAs involved in epigenetic regulatory processes. Also long non-coding RNAs are increasingly perceived as important players in the regulation of gene activity [122] (Figure 3). It is tempting to speculate that *UPG2* and the long non-coding RNA related to *QGJ* might act in target gene regulation by association to ribosomes. The identification of long non-coding RNAs with potential relevance for apomeiosis uncovers a new layer of complexity of regulatory processes. While the molecular mechanisms of their activities have so far not comprehensively been elucidated, involvement of long non-coding RNAs in the control of meiosis appears to be a conserved feature of eukaryotes. Evidence for the importance of non-canonical ORFs in general is given in yeast, where they have high occupancy in meiotic cells [119]. In the fission yeast *Schizosaccharomyces pombe*, the polyadenylated long-non coding RNA meiRNA forms a nuclear body in meiotic cells and is involved in the regulation of the entry into meiosis, homologous pairing and chromosome retention [123]. In plants, evidence for high abundance of long non-coding RNAs in meiocytes comes from a study from sunflowers suggesting their importance during meiosis [124]. Taken together, this provides accumulating evidence for the importance of such regulatory processes controlling RNA abundance and activity for germline specification and likely for discrimination of plant meiosis and apomeiosis. However, experimental prove for this is largely lacking to date. 

Importantly, ribosome assembly and function is typically correlated with the activity of RNA helicases and other types of RNA binding proteins often associated to ribonucleoprotein complexes [125,126] (Figure 3). Members of the large gene family of RNA helicases are involved in basically any aspect of RNA metabolism, storage and degradation. They are of crucial importance for gene regulation to control developmental processes, and they are involved in epigenetic processes. In addition, RNA helicases are central players in transforming stress induced signals into regulatory responses [127]. From studies in sexual *A. thaliana*, the abundant activity of RNA helicases in the MMC has previously been determined, reminding of the crucial and conserved roles of RNA helicases for germline development in animals [107]. Thereby, the RNA helicase *MNEME* (*MEM*) has been uncovered that plays a role to restrict germline fate to allow the specification of only one MMC per ovule [107]. In *A. thaliana* plants carrying a mutant allele of *MEM* frequently AIC-like cells form adjacent to the sexual MMC which give rise to formation of presumably unreduced gametophytes, closely resembling apospory [107]. Furthermore, comparative cell type-specific transcriptome analyses of MMC versus AIC and the cells of the mature embryo sac in sexual *A. thaliana* versus the triploid diplosporous apomicts *Boechera gunnisoniana* point towards a deregulation of *MEM*. However, a consistent differential gene expression in reproductive nucellus tissues prior to apo(meiosis) could not be identified in different sexual and apomictic *Boechera* accessions [80,103]. Given the broad developmental flexibility during apomictic germline formation in *Boechera* [33], a role in regulation of developmental processes relevant for apomixis cannot be ruled out by this finding. Apart from the identification of *MEM*, further studies provide evidence of RNA helicases to be likely involved in regulation of apomictic development, as in *Brachiaria brizantha* and in *Hypericum perforatum*, *BrizHELIC* and a homologue of *MATERNAL EFFECT EMBRYO ARREST29*, respectively, are differentially expressed in tissues of sexual and apomictic plants [10,128]. 

### 5.2. Epigenetic Regulatory Pathways Are Involved in Regulation of Germline Development 

RNA helicases and non-coding RNAs are also players in epigenetic regulatory pathways. Epigenetic regulatory processes which modify gene activity based on DNA methylation, histone modifications, and modulation of chromatin structure are involved in controlling diverse developmental and cell fate decisions. Epigenetic regulation is increasingly recognized to be important during plant germline development [129]. While long non-coding RNAs can act in modulating DNA methylation and histone modifications to regulate gene activity [130], future investigations are required to elucidate their specific importance during reproductive development in detail. 

First evidence for the involvement of epigenetic regulatory pathways in controlling components of apomixis comes from the heterochromatic nature of apomixis loci. Furthermore, alteration in epigenetic regulations might be a consequence of polyploidization and hybridization. It has been hypothesized that apomixis might be superimposed on sexual reproduction by epigenetic control mechanisms. This is suggested from a study in *Paspalum ssp*., where treatment of apomictic plants with the demethylation agent 5′-azacytidine leads to reduction in the frequencies of parthenogenesis [29]. This is in line with the indications for roles of epigenetic regulation for apomixis coming from comparative transcriptional analyses. Also functional evidence supports this notion, as mutations in certain epigenetic regulators lead to induction of elements of apomixis in sexual plants. Studies in *A. thaliana* and maize revealed phenotypes reminiscent of apospory or diplospory for mutants in different players in small RNA and DNA-methylation pathways [131,132,133,134]. This included *ARGONAUTE9* (*AGO9*) and additional genes involved in the RNA directed DNA-methylation pathway [132,134]. AGO proteins act by binding different types of small RNAs, such as microRNAs (miRNA), small interfering RNAs (siRNA), and PIWI-associated RNAs (piRNAs) [135]. Knowledge about their roles in natural apomicts is so far limited.

To gain insights into the possible involvement of small RNAs in apomixis control, their activity has been studied in different natural apomicts including *Eragostris curvula* [136], *Paspalum notatum* [29,137], *Hieracium* subgenus *Pilosella* [119], and *Boechera* [138,139]. Differential representation of small RNA reads in *Paspalum notatum* points towards their involvement in regulation of meiosis, cell cycle control, transcriptional regulation and hormonal signaling [117]. In contrast, in *Hieracium* only small numbers small RNA targets were identified as differentially expressed [114]. From comparisons of sexual and apomictic *Boechera* ovules differential activity of the small RNAs miR156/157 has been identified which is relevant for the regulation of the transcription factor *SQUAMOSA PROTEIN BINDING PROTIEN LIKE 11* (*SPL11*) [139]. As epigenetic regulations are highly dynamic, future studies focusing on elucidating DNA modifications and small RNAs at cell and tissue type-specific resolution will be relevant to gain additional meaningful insights into the contribution of epigenetic regulation to apomixis control. Also, while the investigations on mutant lines of sexual *A. thaliana* and maize indicate that the two modes of reproduction are related, it is currently not well understood how. In the mutants, elements of apomixis establish in sexual species by disabling major players in epigenetic regulatory pathways. This suggests that certain components of apomixis can at least derive from mutations leading to a loss of gene function. However, if apomixis loci epigenetically control the sexual pathway, it remains to be elucidated if suppression of sexual reproduction alone is sufficient for apomixis to establish. 

### 5.3. Cell-Cycle Control and Regulation of Meiosis is Differentially Regulated during Meiosis and Apomeiosis

Apart from mutations in genes involved in epigenetic regulations, also certain mutations or combinations of mutations in core meiotic genes can lead to apomeiosis instead of meiosis. In *A. thaliana* apomeiosis by a diplospory-like mechanism has been observed in plants carrying mutations in *DYAD/SWITCH*. While leading to sterility at high penetrance, also formation of triploid offspring retaining parental heterozygocity occurs at very low frequencies below 1% [140]. Also in maize *ameiotic1* mutants, which is an orthologue of *SWITCH*, designated MMCs undergo a mitosis-like division instead of meiosis [141]. In addition, triple mutants of *sporulation 11-1* (*spo11-1*), *omission of second division 1* (*osd1*), and *recombination 8* (*rec8*) or the A-type cyclin *cyc1;2/tardy asynchronous meiosis* (*tam*) lead to mitotic division instead of meiosis in MiMe1 and MiMe2, respectively [142,143]. This has first been shown for *A. thaliana* and subsequently been used to generate clonal offspring in *A. thaliana* and rice. Clonal offspring has been obtained by combining the meiotic mutants with a manipulation of the centromere-specific histone variant CENH3 that leads to an elimination of the paternal genome [144,145]. Recently, in rice, generation of clonal seeds using MiMe in combination with editing of *MATRILINEAL* has also been demonstrated [146]. *MATRILINEAL* encodes a sperm specific phospholipase and has previously been identified as haploid inducer in maize [147]. 

While these studies provide a prove of concept that engineering of clonal crop plants is feasible, it remains unclear if and how similar mechanisms are involved in natural apomicts. From transcriptional studies strong evidence is provided for differences in regulation of meiosis and cell cycle to play a role during megasporogenesis in sexual and apomictic plants [80,103]. However, it is important to note that a consistent deregulation of core meiotic genes in all studied sexual versus apomictic accessions has not been observed in *Boechera* [103]. From studies of the *Hieracium praeltum* AIC no expression of 14 selected meiotic genes has previously been observed, consistent with the cellular fate destined to mitosis [108]. The lack of consistency likely relates to the developmental flexibility during germline formation: As apospory and diplospory both occur in *Boechera* at different frequencies [33], this also results in different frequencies of the determination and meiosis of the sexual MMC in addition to the AIC. Therefore, future studies focusing on proteins at cellular level will be required to disentangle their involvement in regulation of apomeiosis.

### 5.4. Signal Transduction, Cell-Cell Communication and Hormonal Pathways Appear to Be Involved in Regulation of Apomixis

The developmental flexibility during germline specification and transition to gametogenesis further suggests that involvement of cell-cell communication is required in the regulation of these processes. During sexual reproduction, acquisition of reproductive fate of additional sporophytic cells in the ovule is typically suppressed. As previously determined in maize, rice and *A. thaliana*, this involves the activity of signaling pathways, including the *A. thaliana* Leucine rich repeat receptor kinases *SOMATIC EMBRYOGENESIS RECEPTOR-LIKE KINASE1/2* (*SERK1/2*) [116]. From studies in *Poa pratensis, PpSERK* has been proposed as candidate that is activated in apomictic nucelli to enable development of the apomictically derived gametophyte [10]. The SERK signaling pathway might interact with auxin hormonal pathways involving APOSTART [10]. Strong evidence for the importance of signaling and hormonal pathways for specification of the apomictic germline lineages also comes from transcriptional analyses of sexual and apomictic *Boechera*. From the analysis of genes with evidence of expression in the *B. gunnisoniana* AIC and not in the *A. thaliana* MMC, an enrichment of “MAP kinase kinase” activity was observed in addition to gene and protein families related to auxin transport and signaling [80]. When comparing transcriptional profiles of *Boechera* nucelli harboring MMCs or AICs furthermore the homologue of *A. thaliana GRETCHEN HAGEN3.6* (*GH3.6*) is consistently higher expressed in apomicts as compared to sexual plants [103]. It encodes a GH3 family protein involved in modulation of auxin response. *GH3* was further identified as higher expressed in AICs than in embryo sacs of *Hieracium praealtum*, further suggesting its importance for megasporogenesis in apomicts [109]. Also the homologue of the nonethylene receptor *HISTIDINE KINASE1* (*HK1*) is higher expressed in apomictic as compared to sexual nucelli in *Boechera*. In *A. thaliana* HK1 is involved in abscisic acid signal transduction in response to salt and drought stress [103]. A homologue of HK1 is furthermore located on the *Hypericum* HAPPY-locus. The potential functional relevance of HK1 activity for apomixis has not been described to date. Nevertheless, the reports provide strong evidence for the importance of cell communication and hormonal pathways for apomixis regulation.

### 5.5. Regulation of Seed Development in Apomicts Might Require both Repression and Activation of Gene Activity 

To allow for successful seed development, a precise coordination of development of the embryo, endosperm, and also the seed coat is required. In most species also the maintenance of precise ratios of parental contributions and controlled activation of maternal or paternal alleles for certain regulators of seed development by imprinting is critical [148]. During sexual plant reproduction, double fertilization of the two female gametes (egg cell and central cell) with the two sperm cells initiates formation of the embryo and its nourishing tissue, the endosperm (Figure 3B). It is well understood that double fertilization occurs almost simultaneously [149]. Prior to fertilization, the female gametes are arrested in the cell cycle with the egg cell presumably at G1 of mitosis and the central cell at G2 [150]. At the time of fusion, cell cycle synchronicity between male and female gametes appears critical for initiation of embryo and endosperm development [150]. In sexual species, an increase in Ca^2+^-concentration has been proposed to be the signal for activation of the zygotic program in vertebrates and potentially also in plants [10,151] (Figure 3B). Furthermore, in the sexual mature embryo sac prior to fertilization, the egg cell chromatin is highly condensed and thus in a repressive and transcriptionally silent state [151] (Figure 3B). This might be relevant for the acquisition of potency to allow embryogenesis [151]. However, it might also represent a mechanism to prevent premature or autonomous egg cell activation in the absence of fertilization. 

To allow seed development in apomicts, several aspects of the regulation in sexual species need to be altered. For parthenogenesis, the repressive state of the egg cell needs to be either omitted by precocious activation of embryogenesis or relieved, potentially by chromatin remodeling as the underlying mechanism [151]. Similarly, for autonomous endosperm development, the repression of central cell proliferation needs to be overcome. In sexual species, this repression in the absence of fertilization requires the activity of Polycomb group proteins (Figure 3B). These are interacting in Polycomb Repressive Complexes 2 (PRC2) to control target gene activity by introduction of histone modifications and repressive H3K27me3 marks [152]. In *A. thaliana* mutant alleles of genes encoding components of the MEA-FIE PRC2 complex, in particular *MEDEA* (*MEA*), *FERTILIZATION INDEPENDENT SEED2* (*FIS2*), *FERTILIZATION INDEPENDENT ENDOSPERM* (*FIE*), and *MULTICOPY SUPRESSOR OF IRA1* (*MSI1*), lead to fertilization independent initiation of endosperm development [152]. Implications of FIE for endosperm development have been discussed also for apomictic *Hieracium*; however, the composition of the PRC2 complex including FIE appears to be different from the one identified in *A. thaliana* [153]. 

It is feasible that the coordination of all components of seed development is under dual or more complex control. This likely involves signals keeping the quiet state of the gametes and repressing their development, while other signals are needed to activate the developmental programs. Evidence for such twofold control mechanism is given from the “Salmon system” used for haploid production in wheat by activating autonomous embryogenesis [151]. Thereby two nuclear genes are involved which are the inducer *Parthenogenesis gain* (*Ptg*) under sporophytic control and the repressor *Supressor of Parthenogenesis* (*Spg*) under gametophytic control. This control mechanism appears to be in contrast to findings that activation of BBM and BBM-like genes in the egg cell of rice alone is sufficient to trigger parthenogenesis [75]. However, future investigations and closer understanding of the regulation of *BBM* and *BBML* genes both in sexual and apomictic plants might resolve this puzzle. It is interesting to note that BBM belongs to the group of *APETELA2/ETHYLENE RESPONSIVE FACTOR* (*AP2*/*ERF*) transcription factors. Indications have been found that members of this group are regulated by histone modifications [154]. For the *BBM1* homologue of *Coffea canephora* evidence of epigenetic regulation based on DNA-methylation and histone modifications is given [155]. Strikingly, strong evidence indicates an involvement of H3K27me3 marks in the regulation of *BBM1* [155]. This raises the question, if epigenetic repression based on PRC2 activity and DNA-methylation are responsible for the repression of the maternal allele as observed in rice. This could be a mechanism that safeguards to keep the egg quiet in the absence of fertilization and thus prohibits parthenogenesis to occur in sexual plants. 

It is likely that the molecular machineries controlling development of all components of the seed in different natural apomicts are more complex. In pseudogamous apomicts, which depend on fertilization of the central cell for endosperm development, also parthenogenesis appears to remain repressed in the absence of fertilization as recently shown for *Boechera gunnisoniana* [80] (Figure 3B). The underlying molecular control is likely not involving a fusion of the egg and sperm cells nuclei. In contrast to animals, where sperm dependent parthenogenesis is a common mechanism that still requires fertilization for embryogenesis without paternal contribution [151], similar mechanisms have so far not been observed for plants. From a recent study in apomictic *Boechera*, the second sperm cell nucleus typically does not fuse with the egg cell nucleus [156]. Still the occasional formation of BIII hybrids by fertilization of an unreduced egg cell demonstrates that this fusion is not strictly prevented in the apomicts [43]. Interestingly, differential regulation of CENH3 in sexual and apomictic gametophytes as observed in comparative transcriptional analyses including sexual *A. thaliana* and apomictic *B. gunnisoniana* suggests that this might serve to safeguard embryogenesis without paternal contribution [80]. This would imply that only the maternal genome is retained during early embryogenesis. Similarly, mechanisms of depletion of the paternal genome after occasional fertilization might be reinforced by mechanisms related to the heterochromatic B-like chromosomes in *Boechera*, as previously demonstrated for jewel wasps [157]. In the jewel wasp *Nasonia vitripennis* paternally inherited B-chromosomes promote their own transmission at the expense of other paternal chromosomes which are eliminated to form a haploid embryo [157]. Future studies will be needed to uncover, if similar pathways are active in plants. 

Taken together, current knowledge suggests that complex regulatory networks act upon germline specification and development both in sexual plants and apomicts. The observed developmental flexibility of apomictic reproduction might thereby represent a trait off from the deregulation of developmental programs resulting in incompletely established or leaky mechanisms, however it might also be a mechanism enforcing new evolutionary options. While in different plant systems different candidate genes have been proposed to be relevant for apomixis, common features of regulatory machineries emerge that can serve as a starting point for future functional and evolutionary investigations. Importantly, genetic analyses of apomictic linked loci also consistently indicate a considerable divergence as compared to sexual loci. Thus it cannot be excluded that important regulators of apomixis might so far have been overlooked due to largely restricting the search to described and annotated genes and genomic elements. While due to the heterozygotic or polyploid nature of apomicts genome assembly and annotation remains challenging so far, especially state of the art sequencing technologies increasingly allow to obtain longer sequence reads. In conjunction with further studies focusing on genome evolution in apomicts and related sexual species, this will set the basis for a deeper understanding of the origin of apomixis and its genetic basis.

## 6. Are Stress Signal and Nutritional State Triggering the Determination for Sexual Reproduction or Apomixis?

Sexual reproduction and apomixis are classically viewed as two alternative types of reproduction with their own evolutionary histories. Recent ideas and insights challenge this perception and consider that both reproductive strategies might be polyphenic [18]. In this view, both modes of reproduction can be temporarily activated based on environmental conditions, stress, and nutritional state [18]. Evidence for the potential of a stress induced switch from apomixis to sexuality is given for a number of apomictic systems including *Boechera*, *Paspalum*, *Ranunculus*, and *Eragrostis* [18]. This might resemble an ancient mechanism. It has been hypothesized that the evolution of sexuality is a result of reactive oxygen species (ROS) that were generated starting with the development of primitive mitochondria at the basis of the evolution of eukaryotes [158,159]. From this perspective, the necessity for sex would be the consequence of ROS induced DNA damages as it allows purging of deleterious mutations from the genome [158]. Oxygen based DNA damage might have been also a requirement for meiosis to evolve and thereby lay the foundation for sexual reproduction [160]. It has been hypothesized that interactions of oxidized DNA and the core meiotic gene *SPO11* has enabled double strand breaks and meiotic recombination to occur [160]. Curiously, however, in maize anthers, low levels of ROS promotes acquisition of meiotic fate [161]. From a recent study in *A. thaliana*, repression of the homeobox gene *WUSCHEL* (*WUS*) is important for the acquisition of meiotic fate by the MMC [162]. Relieving the repression of *WUS* activity in the MMC causes mitotic divisions before the cells eventually enter meiosis [162]. Interestingly, in the shoot apical meristem WUS activity is activated by ROS [163]. It might be speculated that also in the maize anthers *WUS* regulation might be involved in determination of meiotic fate in response to ROS levels, if similar to regulatory processes are active in reproductive tissues. Furthermore, ROS has an impact on epigenetic regulatory systems and global DNA-methylation, suggesting the integration of stress signals and epigenetic regulation to control reproduction [18].

Consistent with a role of ROS to trigger meiosis, recent evidence suggests that redox regulation differs in sexual MMCs and the AICs (Figure 3A). In *Boechera gunnisoniana*, enrichment of polyamine and spermidine synthesis is a characteristic feature of the AIC [80]. This is in line with the identification of spermine/spermidine synthase from the ASGR in *Pennisetum squamulatum* [49]. The importance of the polyamine spermidine to protect the DNA from oxidative damage by scavenging of free radicals arising mostly from ROS has long been described [164]. Potentially, the importance of detoxification of ROS in the AIC is a consequence from the absence of meiosis. Such mechanisms to prevent deleterious mutations to arise by oxidative stress appear to be relevant particularly in the founder cells of the apomictic germline lineages. Interestingly, in *Boechera* nucelli tissues an UDP-glycosyltransferase superfamily protein is significantly higher expressed in all sexual as compared to all apomictic accessions analyzed [103]. While the functional role of this gene has not been investigated, it might be involved in synthesis of callose, as shown for certain members of this gene family [165]. Callose deposition is promoted by ROS [166], further supporting the idea that redox stress plays different roles for meiosis and apomeiosis, particularly as callose is typically not enclosing the AIC in contrast to the MMC [167,168] (Figure 3A). Thereby, callose deposition around the MMC might either shield the surrounding cells from ROS and its effects, or might be effective in protection of the meiocyte from disturbances. Future molecular studies are required to shed light onto this question. Nevertheless, the connection of ROS and callose further supports the idea of the importance of redox state for mode of reproduction. 

Apart from ROS other types of stress like nutritional starvation and abiotic stress conditions including cold and heat have a great and versatile impact on meiosis and reproduction. Thereby, not only a shift from apomixis to sexual reproduction occurs, but also alterations of meiosis concerning recombination frequencies or the formation of unreduced or aneuploid gametophytes [169]. A central integrator of nutrient, energy, and stress related signals to regulate cell growth and development in eukaryotes is the target of rapamycin (TOR) kinase. In the yeast *Schizosaccharomyces pombe* nutritional starvation triggers the onset of meiosis and sexual reproduction dependent on the activity of TOR pathways [170]. Recent evidence suggests an evolutionary conservation of these pathways, as application of glucose at a certain developmental time point leads to features of apomeiosis in sexual *A. thaliana* [171]. The underlying molecular mechanism remains to be investigated in detail. An interesting question will be if *WUS* activity in the MMC is elevated by application of glucose, a mechanism described for the shoot apical meristem [172]. If so, this might be the molecular mechanism of obtaining mitotic divisions of the MMC similar to previous reports on de-repression of WUS activity in the MMC [162].

Interestingly the TOR pathway also coordinates ribosome activity [173], implying a connection between stress, nutritional state, reproduction and cell cycle. Further ribosome biogenesis factors like RNA helicases are not only involved in the regulation of gene activity and developmental decisions, but also in mediating stress response and growth regulation [126]. Functional implications in stress response have in particular been described for a number of RNA helicases, including *AtRH36* involved in regulation of gametogenesis and *ENHANCED SILENCING PHENOTYPE3* that has previously been described to be active in the AIC in *Boechera gunnisoniana* unlike in the *A. thaliana* MMC [80,126]. Also heat shock proteins are stress responsive proteins tightly associated to ribosome function, as they typically assist folding of newly derived polypeptide sequences to proteins as chaperones. Evidence for roles of heat shock proteins in apomixis regulation comes from different types of apomixis, including apospory in *Hieracium prealtum* [34], apomixis in *Paspalum notatum* [92], somatic embryogenesis in *Citrus* [76], and apogamy in the fern *Drypteris affinis* [174]. In addition, it is interesting to note that AP2/ERF transcription factors are important players in the integration of hormonal pathways and stress responses to control developmental decisions [154]. 

The regulation of reproductive development related to environmental factors and nutrition represents a conserved mechanism in eukaryotes. It can easily be envisioned that particularly in largely facultative apomictic systems such factors allow us to modify the frequencies of apomixis. Nevertheless, the heritability of apomixis and the identification of the genetically linked loci suggests the requirement of certain genetic elements for apomixis.

## 7. Brief Summary and Conclusions

Despite longstanding interest in apomixis, the gene regulatory processes and molecular mechanisms underlying apomixis are currently not fully understood. It remains an unresolved puzzle, how all major components of apomixis derived simultaneously several times independently. Recently, alternative concepts are discussed proposing the possibility of sexual reproduction and apomixis as ancient alternatives. In this view, conserved molecular machineries control the mode of reproduction dependent on nutritional state and environmental conditions. Irrespective of its evolutionary origin, apomixis is characterized by distinctive changes in the gene regulatory program as compared to sexual reproduction. Increasing evidence suggests that interrelated regulatory control mechanisms are involved, including epigenetic regulatory pathways, cell cycle control, regulation of protein turnover and degradation, signal transduction pathways, and hormonal regulatory pathways. Particularly for (apo)meiosis the assembly and regulation of ribosomes and associated factors emerge as novel important layer of regulation. Furthermore, precise coordination of repression and activation of gene activity appears to be involved in the transition from the female gametes to embryo and endosperm development. Thereby, BBM and BBML proteins have emerged as important players to promote embryogenesis. Taken together, recent insights into the molecular mechanisms and genetic basis underlying apomixis provide an important basis for future investigations directed on the detailed understanding of the regulatory programs involved. One important focus should be on fundamental and evolutionary conserved mechanisms to modify gene and protein activity and on the impact of environmental conditions on reproductive mode and success. This will not only allow us to gain fundamental new insights in the developmental processes of reproduction, but will also be an important basis for the harnessing of plant reproduction and apomixis for agricultural applications. 

## Figures and Tables

**Table 1:** Candidate genes for apomixis encoded from apomixis linked loci.

Gene	Type of Apomixis	Element of Apomixis	Locus	Plant Family	Species	Publication
*ARI7*	gametophytic	apospory	HAPPY	*Hypericaceae*	*Hypericum perforatum*	[44,62]
*APOLLO*	gametophytic	female apomeiosis	-	*Brassicaceae*	*Boechera ssp.*	[63,64]
*UPG2*	gametophytic	male apomeiosis	-	*Brassicaceae*	*Boechera ssp*.	[65]
*QGJ*	gametophytic	apospory	-	*Poaceae*	*Paspalum notatum*	[67]
*ORC3*	gametophytic	endosperm formation	ACL	*Poaceae*	*Paspalum simplex*	[51,68]
*BBM*(*L*)	gametophytic	parthenogensis	ASGR	*Poaceae*	*Pennisetum squamulatum* *Cenchrus ciliaris* *Brachiara decumbens*	[49,69,70,73,74]
*RKD*	sporophytic	somatic embryogenesis	-	*Rutaceae*	*Citrus*	[5,76]

**Table 2:** Transcriptional analyses on reproductive tissues to identify genes involved in apomixis regulation.

Plant Family	Species	Type of Apomixis	Tissues Profiled	Methods of Analysis	References
*Poaceae*	*Pennisetum ciliare*	gametophytic	unpollinated ovaries	modified differential display	[83]
*Poaceae*	*Pennisetum glaucum*	gametophytic	spikelets at 4 developmental stages (pre-meiosis, meiocyte, gametogenesis, mature gametophyte)	suppression subtractive hybridization	[84]
*Poaceae*	*Panicum maximum*	gametophytic	flower buds	cDNA library	[85]
*Poaceae*	*Panicum maximum*	gametophytic	spikelets (pre-meiosis)	RNA-Seq (Illumina HiSeq2500)	[86]
*Poaceae*	*Panicum maximum*	gametophytic	immature pistils	custom microarray	[110]
*Poaceae*	*Paspalum notatum*	gametophytic	inflorescences at 4 developmental stages (early premeiosis; late premeiosis/ meiosis; postmeiosis; anthesis)	RNA-Seq (Roche 454)	[94]
*Poaceae*	*Paspalum notatum*	gametophytic	florets at different developmental stages	cDNA-AFLP	[92]
*Poaceae*	*Paspalum notatum*	gametophytic	inflorescences	differential display analysis	[91,111]
*Poaceae*	*Eragostris curvula*	gametophytic	panicles	differential display analysis	[112]
*Poaceae*	*Eragostris curvula*	gametophytic	spikelets with embryo sacs at all developmental stages	RNA-Seq (Roche 454)	[95]
*Poaceae*	*Poa pratensis*	gametophytic	florets at 4 developmental stages (pre-meiosis; meiosis; post-meiosis; anthesis)	cDNA-AFLP	[87,88]
*Hypericaceae*	*Hypericum perforatum*	gametophytic	nucellus tissues harboring MMC or AIC before (apo)meiosis	RNA-Seq (Illumina NextSeq500)	[62]
*Hypericaceae*	*Hypericum perforatum*	gametophytic	pistils	custom microarray	[98]
*Hypericaceae*	*Hypericum perforatum*	gametophytic	whole flowers at range of developmental stages	cDNA libraries	[113]
*Asteraceae*	*Hieracium praealtum* *Hieracium aurantiacum*; *parthenogenesis incapable accession lop138*	gametophytic	ovules and ovaries at different developmental stages isolated by manual microdissection	RNA-Seq (Illumina HiSeq2000)	[100]
*Asteraceae*	*Hieracium praealtum*	gametophytic	AIC, developing female gametophytes (2–4 nucleate), and somatic ovule cells isolated by LAM	RNA-Seq (Roche 454; Illumina HiSeq2000)	[108,109]
*Asteraceae*	*Hieracium praealtum*	gametophytic	ovaries	RNA-Seq (Illumina HiSeq2000)	[114]
*Urticaceae*	*Boehmeria tricuspis*	gametophytic apomixis	Flowers at 4 developmental stages (MMC; FMS, embryo sac, mature embryo)	RNA-Seq (Illumina HiSeq4000)	[88]
*Brassicaceae*	*Boechera*	gametophytic	nucellus tissues harboring MMC or AIC isolated by LAM	RNA-Seq (Illumina NextSeq500)	[103]
*Brassicaceae*	*Boechera gunnisoniana*	gametophytic	AIC, egg cell, central cell, synergids isolated by LAM	ATH1 microarray, RNA-Seq (SOLiD V4)	[81]
*Brassicaceae*	*Boechera*	gametophytic	ovules isolated by manual microdissection	RNA-Seq (Roche 454); custom microarray	[63]
*Brassicaceae*	*Boechera*	gametophytic	antherheads at pollen mother cell stage	custom microarray	[65]
*Brassicaceae*	*Boechera*	gametophytic	ovules isolated by manual microdissection at 4 developmental stages (early premeiosis; late premeiosis; FMS, gametophyte)	SuperSAGE	[101]
*Brassicaceae*	*Boechera*	gametophytic	ovules isolated by manual microdissection pooled flower stages	SuperSAGE; RNA-Seq (Roche 454)	[102]
*Fabaceae*	*Medicago sativa*	apomeiotic mutant	flower buds at 4 developmental stages (pre-meiosis, initial meiosis, final meiosis, and post-meiosis)	cDNA-AFLP	[96]
*Rutaceae*	*Citrus*	somatic embryogensis	fruits 15, 30, 45, and 60 d after flowering	custom microarray	[76]
*Rutaceae*	*Citrus*	somatic embryogensis	leaves, ovules, seeds, fruits	RNA-Seq (Illumina Genome Analyzer)	[5]
*Rutaceae*	*Citrus*	somatic embryogensis	ovaries at anthesis and at 3, 7, 17, 21, and 28 d after flowering	RNA-Seq (Illumina Genome Analyzer)	[115]
